# Propofol and Clevidipine-induced Hypertriglyceridemia

**DOI:** 10.7759/cureus.3165

**Published:** 2018-08-20

**Authors:** Harleen Kaur, Premkumar Nattanamai, Kathryn E Qualls

**Affiliations:** 1 Neurology, Univeristy of Missouri, Columbia, USA; 2 Neurology, University of Missouri, Columbia, USA; 3 Pharmacy, University of Missouri, Columbia, USA

**Keywords:** hypertriglyceridemia, acute pancreatitis, propofol, clevidipine, neurocritical care setting

## Abstract

Hypertriglyceridemia and related pancreatitis due to the use of lipid emulsions such as propofol has been documented, but less is known about the additive adverse effects of propofol and clevidipine lipid emulsions in the literature. We report an unusual case, highlighting the trend of serum triglyceride and pancreatic enzymes (amylase/lipase) with the administration of propofol and clevidipine for a prolonged period in the neurocritical care setting. We present a case of a 27-year-old male who was admitted to the neuroscience intensive care unit (NSICU) for management of severe subarachnoid hemorrhage (SAH) with six-millimeter (mm) midline shift to the left from the rupture of anterior communicating artery aneurysm. The patient was given propofol infusion to maintain sedation and manage intracranial pressures, and clevidipine was chosen over other antihypertensive class for blood pressure management secondary to renal impairment. To focus on the risk of hypertriglyceridemia and associated pancreatitis with the combined use of lipid emulsions we quantified the effect of lipid emulsions on serum triglycerides. We calculated the total calorie and fat content the patient received from the propofol and clevidipine along with the calorie intake from enteral nutrition (Fibersource® tube feed). The patient received a total propofol infusion of 44,391.2 milligrams (mg) over 16 days which accounts for 4,882.99 kilocalories (kcal) and 443.91 grams of fat. He received a total clevidipine infusion of 297 mg over the 48-hour period which contributes 594 kcal and 59.4 grams of fat. The required daily calorie intake through enteral nutrition of Fibresource® was titrated to a goal of 80 mL/hour which provided 2,304 kcal and 76.8 grams of fat each day. We also graphically depicted the rise in the serum triglyceride level after continuous infusion of propofol and clevidipine and subsequent improvement in the amylase and lipase level after the propofol was discontinued. Hence we conclude, careful and periodic monitoring of the serum triglyceride levels and limitation on the total calories from other fat sources such as enteral nutrition can help to mitigate the drug-induced effects.

## Introduction

Propofol is widely used as the sedative-hypnotic agent of choice in the critical care setting because of its potential effect in reducing the mortality and duration of stay in the hospital [1]. It not only acts as a potent anesthetic agent but is also effective in reducing the intracranial pressures and acts as a potential anxiolytic, antiepileptic, neuroprotective agent [2-4]. It mainly acts on the GABA-A (gamma-aminobutyric acid) receptors and increases the GABA activity [2]. Propofol (2,6 diisopropylphenol) is a lipid emulsion with an empiric formula of C_12_H_18_O and consists of 1% or 2% w/v (weight/volume) propofol, 10% soybean oil, 12% egg phospholipid, and 2.25% glycerol.

Clevidipine is an ultrashort-acting lipid emulsion with a half-life of one minute and is used extensively as an antihypertensive of choice in patients with worsening renal function [5]. It acts as a dihydropyridine calcium channel blocker which helps in blood pressure management by reducing the arteriolar resistance. It is highly plasma protein bound and rapidly metabolized via hydrolysis by esterases in the blood; therefore, hepatic or renal dysfunction does not affect its elimination [6]. It is a lipid emulsion formulation containing 0.2 grams/mL of fat (2 kcal/mL) constituted by 20% soybean oil, glycerin (2.25 mg/mL) and purified egg yolk phospholipids (12 mg/mL). Prolonged use of these lipid emulsions in critical care setting can increase the risk of hypertriglyceridemia and associated pancreatitis, which we highlighted in this case report by monitoring the effect of these lipid emulsions on serum triglyceride, amylase, and lipase enzymes.

## Case presentation

**Figure 1:**
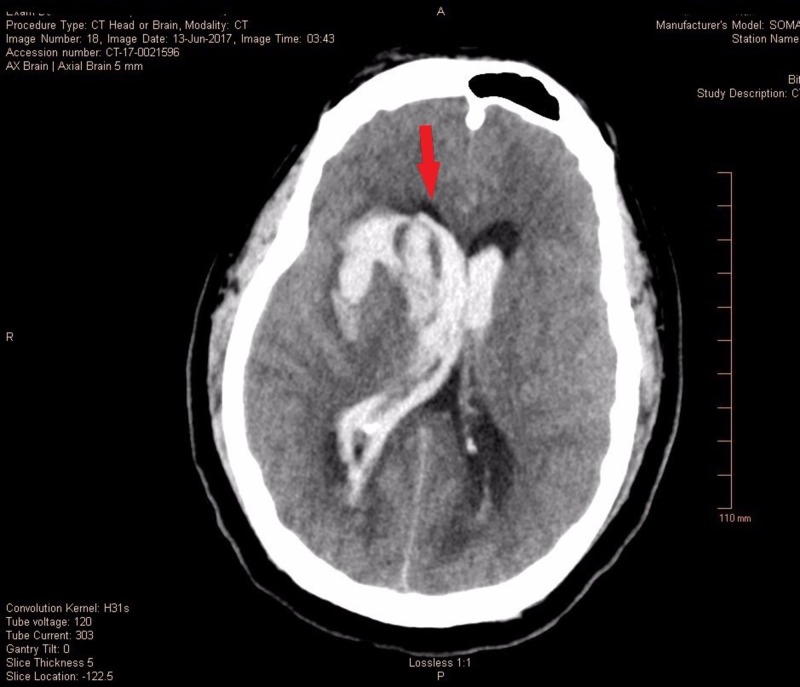
Severe subarachnoid hemorrhage with interventricular extension and midline shift.

**Figure 2:**
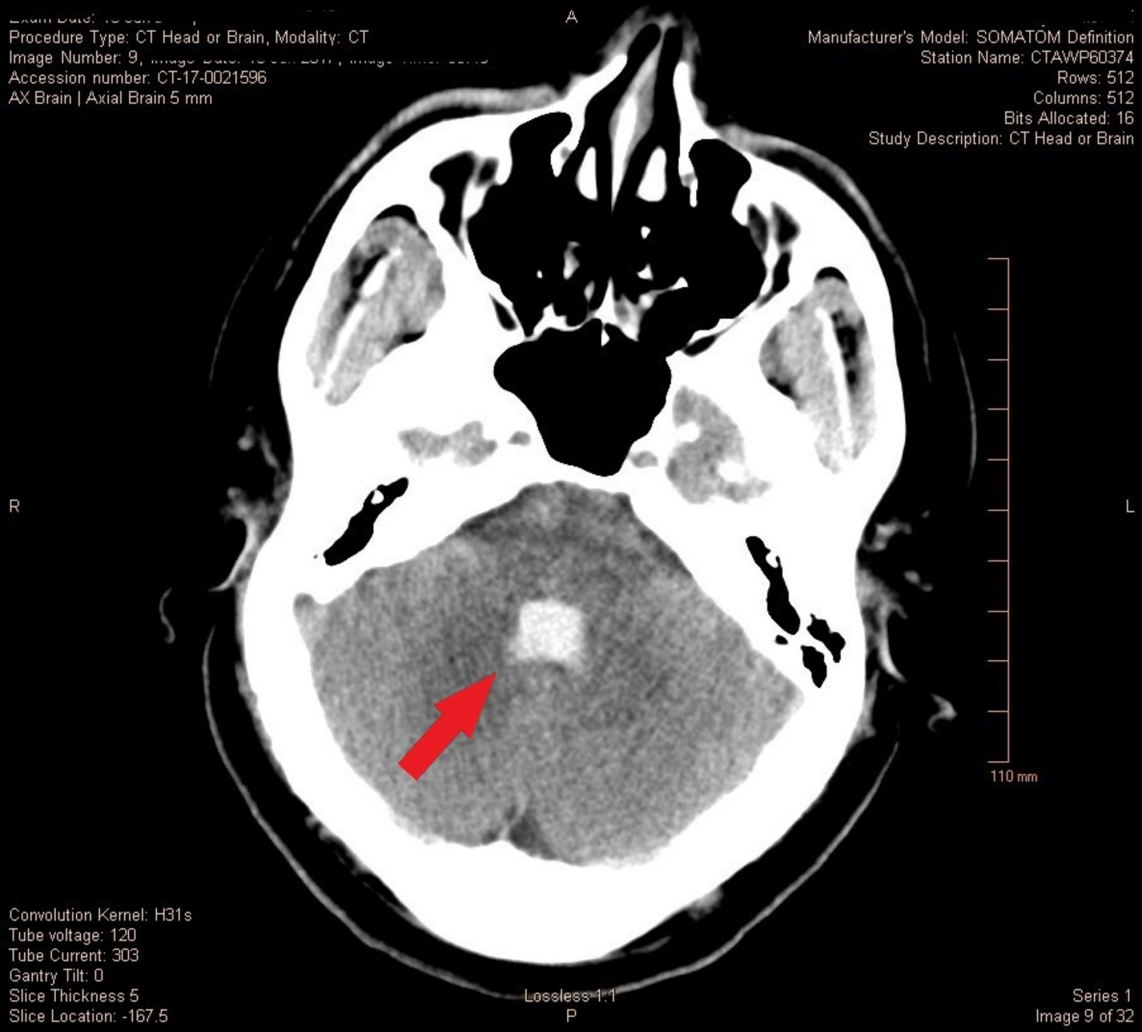
Casting of fourth ventricle with brainstem compression.

A 27-year-old otherwise healthy male weighing 112 kilograms was brought to the neurosciences intensive care unit (NSICU) for the management of a diffuse subarachnoid hemorrhage (SAH) secondary to aneurysm rupture with a Glasgow Coma Scale (GCS) of three, Hunt and Hess Grade 4 and Fischer Grade 5. The non-contrast computed tomography (CT) scan showed diffuse cerebral edema, SAH, intraparenchymal hemorrhage with interventricular extension along with 6 mm of leftward midline shift (Figure 1) with obstructive hydrocephalus and brainstem compression (Figure 2). The computed tomography angiography (CTA) of the head and neck showed 4.0 x 2.3 x 2.8 mm saccular anterior communicating artery aneurysm which was managed by coil embolization. Ventriculostomy was done for impeding obstructive hydrocephalus with the placement of an extraventricular drain (EVD).

The hospital course was complicated by intracranial hypertension which was managed with additional agents including fentanyl, cisatracurium and propofol infusions. On day one, propofol infusion was started on 10 mcg/kg/min (microgram/kilogram/minute) and titrated by 5 mcg/kg/min every five minutes to a maximum of 80 mcg/kg/min. The cerebral edema was managed with the use of mannitol and 3% hypertonic saline; the monitoring goals for this patient were sodium of 150 to 155 mEq/L, intracranial pressure (ICP) of less than 20 mmH20 and cerebral perfusion pressure (CPP) of 60 to 70 mmHg. The first triglyceride level was drawn on day two, with a result of 330 mg/dL.

In the due course, the patient developed poor renal function secondary to contrast nephropathy or mannitol-induced acute kidney injury. His creatinine increased to 1.84 mg/dL and creatinine kinase increased to 703 U/L. On day three, clevidipine was started as the antihypertensive agent over other intravenous antihypertensive class due to acute kidney injury. On day four, a repeat triglyceride level was noted of 527 mg/dL. In concern of the rising triglycerides, clevidipine was changed to labetalol 200 mg three times a day for blood pressure management and the enteral nutrition infusion was reduced to 20 mL/hr to compensate for additional calories from the propofol infusion.

On day six, the patient’s triglycerides had reduced to 240 mg/dL after discontinuing the clevidipine on day four (Table 1). On days five and six the propofol infusion was minimized but then titrated up for increased ICP on day eight and continued at similar rates on day nine. On day ten, his amylase increased to 204 U/L, and lipase increased to 493 U/L. Propofol was discontinued for the next two days (day eleven and twelve), and a fall in the subsequent amylase and lipase levels was noted. Propofol was restarted for ICP management on day thirteen and continued through day sixteen. The average infusion rate of propofol each day is noted in Table 1. Further improvement in pancreatic enzymes was seen after stopping the propofol infusion on day sixteen. Also, the liver enzymes (aspartate aminotransferase (AST)/alanine transaminase (ALT)) was noted to be on the higher side of the normal range, throughout the stay in the hospital (Table 1).

**Table 1:** Data highlighting the trend of serum levels of triglycerides, pancreatic enzymes, liver enzymes with propofol and clevidipine infusion. AST: Aspartate aminotransferase; ALT: Alanine transaminase.

Day Number:	1	2	3	4	5	6	7	8	9	10	11	12	13	14	15	16	17
Clevidipine (mg/day)			129	168													
Propofol (mg/day)	1897.7	4451.3	4482	4838.4	1948.8	3292.8	2453.2	4536	4972.8	3655.8			1814.4	1881.6	3225.6	940.8	
Propofol Average Infusion Rate (mcg/kg/min)	28.2	33.1	27.8	20	26.4	28.8	16.6	28.2	30.8	28.6			22.5	20	20	20	
Calories from Propofol and Clevidipine (kcal/day)	208.7	489.6	1009	1204.2	214.4	362.2	269.9	499	547	402.1			199.6	207	354.8	103.5	
Lipid from Propofol and Clevidipine (g/day)	18.98	44.51	96.42	115.58	19.49	32.93	24.53	45.36	49.73	36.56			18.14	18.82	32.26	9.41	
Serum Triglyceride (mg/dL)		330		527		240	286			469	256	219		231	238	242	196
Serum Amylase (U/L)										204	118	79				68	
Serum Lipase (U/L)						40				493	206	96				76	
AST (U/L)				49		32	38	30		69				156		78	
ALT (U/L)				60		112	104	83		140				207		266	

The patient was started on enteral nutrition with Fibersource. The enteral nutrition was titrated up to 80 mL/hr which accounted for a total daily calorie intake of 2,304 kcal and total fat of 76.8 grams each day. The patient received a total propofol infusion of 44,391.2 mg over 16 days which accounts for 4,882.99 kcal and 443.91 grams of fat. Total clevidipine infusion of 297 mg over the 48-hour period contributed 594 kcal and 59.4 grams of fat (Table 2).

**Table 2:** Estimation of total calories and lipid content from propofol, clevidipine and enteral nutrition.

	Average Calories per Day of Therapy (kcal/day)	Average Lipids per Day of Therapy (grams/day)	Total Calories Day 1 to Day 16 (kcal)	Total Lipids Day 1 to Day 16 (grams)
Propofol	348.78	27.74	4882.99	443.91
Clevidipine	594	59.4	1188	118.8
Fibersource Enteral Nutrition	2304	76.8	36,864	1228.8

On subsequent physical exam, he started following commands including opening eyes, showing two fingers, and wriggling toes. The patient also had an intact cough, gag, and oculocephalic reflex and additionally withdrew to noxious stimuli. His further management included placement of tracheostomy and percutaneous endoscopic gastrostomy with transfer to a long-term acute care hospital after discharge.

## Discussion

**Figure 3:**
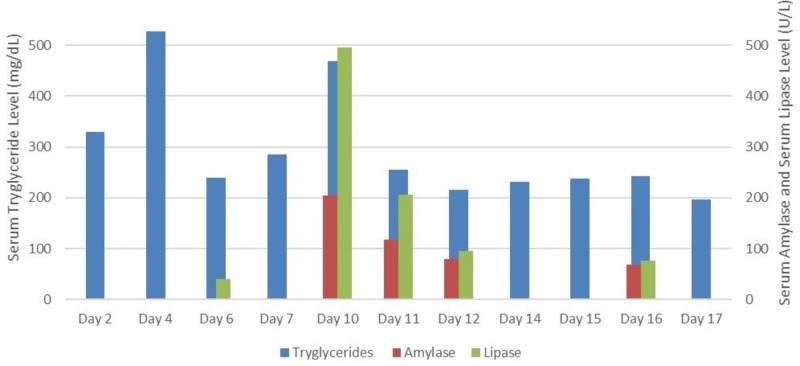
Trend of serum triglyceride, amylase, lipase over the total hospital stay.

Most studies have highlighted the effect of propofol on serum triglycerides or PRIS (Propofol Related Infusion Syndrome) [7,8]. We noted the effect of continuous use of lipid emulsions on serum amylase, lipase, and triglycerides levels. A rising trend in serum triglycerides was noted with the concomitant use of propofol and clevidipine infusion on day four. After discontinuing the clevidipine on day five, fall in serum triglycerides was noted to 240 mg/dL. On day ten, a rising trend in amylase and lipase was seen causing possible pancreatitis from continuous use of lipid emulsions. Propofol was discontinued for the next two days which resulted in improvement in amylase and lipase level. On withholding the propofol on day sixteen, a downward trend was noted in serum pancreatic enzymes level (Figure 3). These trends are further explained graphically in Figure 3, highlighting the fluctuation in serum amylase, lipase, and triglyceride level during the course of hospital stay, from day two when the serum triglyceride level was noted first till day seventeen when final triglyceride level was noted after discontinuing propofol (Figure 3).

**Figure 4:**
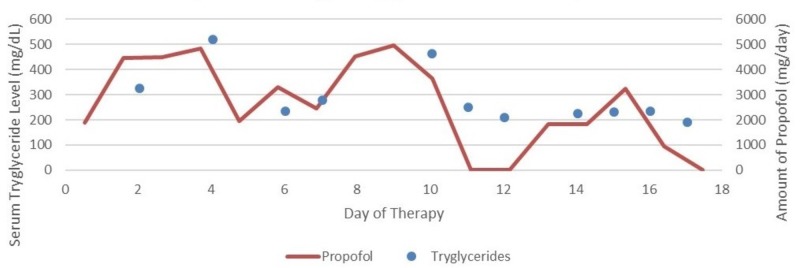
Effect of propofol on serum triglyceride level.

Hypertriglyceridemia and associated pancreatitis is a common adverse effect seen in patients receiving propofol for an extended period. Devaud et al. in their study on 220 patients receiving propofol infusion and nutrition lipid, proposed that 99 patients (45%) presented with hypertriglyceridemia and propofol had the highest correlation to serum triglycerides as compared to nutrition lipids (r(2) = 0.28 and r(2) = 0.26, respectively) [7]. Increase in the cumulative dose of propofol can cause hypertriglyceridemia associated with it (Figure 4). Excessive dose of lipid emulsion does not hydrolyze in the plasma resulting into increase in triglyceride in blood [9].

Periodic checks of the pancreatic enzymes and triglyceride level can help to monitor the potential adverse effects associated with the drug. The concept is favored by the retrospective study performed by Devlin et al. on 159 patients which showed that 29 patients (18%) developed hypertriglyceridemia (>400 mg/dL) out of which 6% developed severe hypertriglyceridemia (>1000 mg/dL), and three (10%) developed pancreatitis (amylase >=125 U/L and lipase >=60U/L). Hypertriglyceridemia was noted at the median propofol infusion of 50 mcg/kg/min in this study [10].

**Figure 5:**
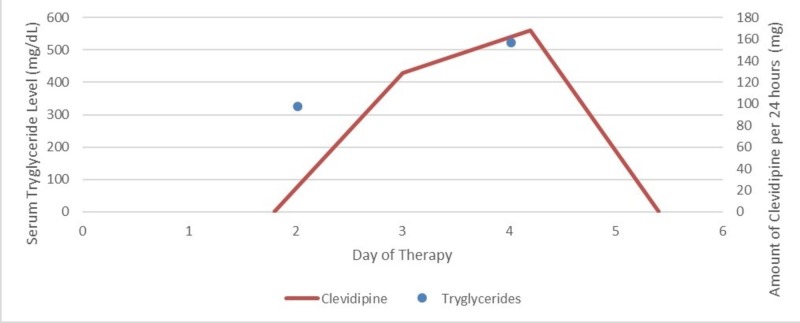
Effect of clevidipine on serum triglyceride level.

Clevidipine is another lipid emulsion with a deleterious effect on serum triglycerides used commonly in the critical care setting. Figure 5 shows the graphical presentation of rising serum triglycerides with the use of clevidipine from day two till day four of the hospital stay (Figure 5). Use of clevidipine as the drug of choice in hypertension management was highlighted by Graffagnino et al. in their study on 35 patients with Intracerebral Hemorrhage, with median GCS score of 12 and mean systolic blood pressure (SBP) of 186 mmHg showed that target blood pressure was maintained within 30 minutes of clevidipine infusion [11]. Since the benefit outweighed the risk in our patient (as he had poor renal function test), clevidipine was chosen as the antihypertensive drug of choice until his renal functions improved.

Our patient had a serum triglyceride of 330 mg/dL at the time of admission to the hospital which might be secondary to excessive alcohol consumption or underlying disorder of deranged pathological lipid metabolism causing hypertriglyceridemia. Excessive consumption of alcohol can lead to an increase in serum triglyceride level by inhibition of lipoprotein lipase activity and increase synthesis of very low-density lipoprotein (VLDL) in the liver [12]. The effect of alcohol consumption on serum triglycerides was explained by Veenstra et al. in their study on 16 healthy men of age group (20-30 year and 45-55 year) proposing that serum triglyceride level measured one hour after alcohol consumption (30 g of alcohol in wine), after a standard dinner could increase by 11.5% (p = 0.044) [13].

Our patient received his nutrition from Fibresource® tube feed (1.2 kcal/ml) which provided with 2304 kcal of baseline calories and total fat of 76.8 g/day (40 mg/ml). In addition to this, he received a total calorie of 4,587.82 Kcal (352.87 kcal/day) and total fat of 415.51 g (0.1 g fat/ml) over a period of 13 days from cumulative propofol dose of 41,707.5 mg. Total clevidipine dose of 353 mg over 48 hours provided extra calories of 1412 kcal and lipid content of 141.2 g fat. Total calories from both propofol and clevidipine came to an average of 1211.2 kcal/day (average).

The use of intravenous fat emulsions has increased amount of free phospholipids which interfere with the lipoprotein lipase activity resulting in the increase in serum triglyceride level with possible pancreatitis [14]. Hence, to reduce the adverse effect, the dose of the lipid emulsions should be administered at a low rate along with the periodic check on serum triglycerides, and pancreatic enzymes to monitor the drug-induced side-effects [15,16].

## Conclusions

Our case report highlights the adverse effect of prolonged use of propofol and clevidipine on serum triglyceride and pancreatic enzyme. Careful monitoring of the tube feeds, regulating the total lipids and dose of lipid emulsions with periodic checks on the serum triglyceride levels can help to overcome drug-induced adverse effect.
